# Urinary ^1^H-NMR Metabolomics Highlights MIIA (Microbiota–Immune–Inflammation Axis) Activation by Organic Mediterranean Diet

**DOI:** 10.3390/metabo15090571

**Published:** 2025-08-26

**Authors:** Laura Di Renzo, Simona Cesaroni, Giulia Frank, Barbara Pala, Daniel Oscar Cicero, Paola Gualtieri, Greta Petrella

**Affiliations:** 1 Section of Clinical Nutrition and Nutrigenomics, Department of Biomedicine and Prevention, University of Rome “Tor Vergata”, Via Montpellier 1, 00133 Rome, Italy; laura.di.renzo@uniroma2.it; 2 Department of Chemical Sciences and Technologies, University of Rome “Tor Vergata”, Via della Ricerca Scientifica, 00133 Rome, Italy; cesaroni@alumni.uniroma2.eu (S.C.); cicero@scienze.uniroma2.it (D.O.C.); 3 PhD School of Applied Medical-Surgical Sciences, University of Rome “Tor Vergata”, Via Montpellier 1, 00133 Rome, Italy; giulia.frank@ymail.com (G.F.); barbara.pala93@gmail.com (B.P.); 4 School of Specialization in Food Science, University of Rome “Tor Vergata”, 00133 Rome, Italy

**Keywords:** mediterranean diet, organic food, metabolomics, exposome, microbiota–immune–inflammation axis

## Abstract

Background: While the Mediterranean diet is well-established for its health benefits, the specific influence of organic versus conventional food sources within this pattern remains underexplored at the systemic metabolic level. Objective: This study investigated the metabolic effects of two matched Mediterranean diets, one based on organically produced foods (IMOD) and the other on conventionally produced equivalents (IMNOD), to assess the impact of food production methods on host metabolism and immune-inflammatory balance. Methods: Twelve healthy adults completed a crossover dietary intervention including IMOD and IMNOD phases. Urinary metabolite profiles were assessed via ^1^H-NMR spectroscopy across 42 compounds. Multivariate and univariate analyses evaluated metabolic responses. Results: Both interventions normalized some out-of-range urinary metabolites. However, IMOD elicited broader and more significant changes, including increased levels of tricarboxylic acid (TCA) intermediates (e.g., isocitrate, trans-aconitate), plant-derived metabolites (e.g., trigonelline), and host–microbiota co-metabolites (e.g., N-phenylacetylglutamine, 1-methylnicotinamide). Simultaneously, fermentation-associated and xenobiotic-linked metabolites such as formate, acetate, and 2-furoylglycine decreased. These shifts collectively represent a beneficial modulation of the Microbiota–Immune–Inflammation Axis (MIIA effect). Conclusions: Organic food consumption within a Mediterranean framework promotes host–microbiota metabolic interplay and enhances immune-supportive biochemical pathways. The findings provide new mechanistic insight into how food production quality contributes to systemic metabolic health and support broader efforts to make organic foods more accessible.

## 1. Introduction

The Mediterranean Diet is widely recognized for its protective effects against chronic-degenerative diseases, including cardiovascular, metabolic, and inflammatory conditions [1,2]. Its distinctive features, such as high intake of plant-based foods, moderate consumption of fish and dairy, and low intake of red meat, promote increased microbial biodiversity, antioxidant status, and overall metabolic resilience [3,4].

In recent years, increasing attention has been paid to the concept of the human exposome, which encompasses the totality of environmental exposures, including dietary xenobiotics such as pesticides, additives, and heavy metals, that interact with genetic and physiological systems to influence health outcomes [5]. Diet is a primary component of the human exposome—the totality of environmental exposures that interact with our biology across the lifespan. Among these, chronic ingestion of synthetic chemicals commonly present in conventionally produced foods (such as pesticide residues, additives, and processing by-products) has been linked to alterations in gut microbiota composition, low-grade systemic inflammation, and an elevated risk of chronic non-communicable diseases [6,7,8,9]. In contrast, organic food consumption significantly reduces pesticide intake, thereby contributing to a lower toxic burden and supporting long-term health promotion [10,11,12]. Furthermore, the Microbiota–Immune–Inflammation Axis (MIIA) encompasses the complex bidirectional interactions between gut microbes, the host immune system, and inflammatory pathways. Commensal bacteria are essential for the maturation of both innate and adaptive immunity, influencing epithelial barrier integrity, T-cell regulation (including Treg/Th17 balance), and systemic inflammatory tone [13,14,15]. Perturbations of this axis, due to diet, xenobiotic exposure, or microbial imbalance, can disrupt mucosal homeostasis and contribute to chronic-degenerative disease risk.

Within the Mediterranean Diet’s framework, organically produced foods may further reinforce its beneficial effects. Organic crops are typically richer in polyphenols and antioxidants [16,17], and their integration into Mediterranean Diet-based interventions has been associated with improved metabolic and inflammatory profiles [18]. For instance, a short-term Italian Mediterranean Organic Diet (IMOD) has previously been shown to reduce circulating levels of homocysteine and phosphorus in both healthy subjects and nephropathic patients. The reduction in homocysteine levels following the IMOD intervention may be attributed to improved intake of B-group vitamins (B6, B9, and B12), which are essential cofactors in homocysteine metabolism. Deficiencies in these vitamins impair homocysteine clearance and are linked to increased cardiovascular risk [19]. Organic diets, typically richer in unprocessed plant foods, may offer higher levels of these micronutrients. Similarly, the observed decrease in phosphorus may reflect reduced intake of phosphate additives, which are more prevalent in conventionally processed foods and associated with vascular burden [18].

A previous study demonstrated that IMOD, compared to its conventionally sourced counterpart (Italian Mediterranean Non Organic Diet—IMNOD), led to more favorable modulations of the gut microbiota, particularly increasing the abundance of beneficial taxa involved in short-chain fatty acids (SCFAs) production and intestinal homeostasis [16]. These findings suggest that food quality, beyond macronutrient distribution, plays a critical role in shaping host-microbiota interactions and promoting systemic health.

Advanced technologies such as metabolomics offer a powerful approach to characterize the metabolic impact of dietary exposures in a holistic and unbiased manner. By capturing a wide array of endogenous and exogenous metabolites in biofluids, metabolomics enables the assessment of biochemical changes induced by food components, including polyphenols, fatty acids, and contaminants [17]. This approach is particularly relevant in the context of organic versus conventional food consumption, as it allows for the identification of metabolic pathways modulated by food quality and production practices.

Recent studies have demonstrated that the metabolomic fingerprint of individuals consuming organic diets is distinct, with shifts in metabolites related to oxidative stress, inflammation, and lipid metabolism [20]. Integrating metabolomics into nutritional studies thus provides mechanistic insight into how dietary patterns influence human health beyond conventional nutrient analysis, supporting a more personalized and exposure-informed framework for dietary recommendations.

Among the various analytical platforms used in metabolomics, proton nuclear magnetic resonance (^1^H-NMR) spectroscopy has emerged as a robust and widely applied technique for the untargeted analysis of biofluids, especially urine [21]. Its key advantages include high reproducibility, non-destructive measurement, minimal sample preparation, and the ability to detect a broad range of both endogenous and exogenous metabolites in a single run [22]. In the context of nutritional interventions, ^1^H-NMR is particularly valuable for longitudinal and crossover designs, as it enables quantitative comparisons across timepoints and conditions [23,24].

Leveraging this analytical approach, this study aimed to investigate the metabolic responses triggered by two Mediterranean dietary patterns that were compositionally similar but differed in their food production methods, organic versus conventional. The goal was to assess how food quality alone can influence human metabolism.

## 2. Materials and Methods

### 2.1. Study Design

This study was conducted as a randomized, longitudinal, crossover-controlled trial to investigate the impact of food production systems (organic vs. conventional) within the context of an Italian Mediterranean Diet. The trial took place at the University of Rome ‘Tor Vergata’ between 1 June and 31 October 2024.

Eligibility criteria included: adults aged 18–65 years; absence of chronic gastrointestinal diseases (e.g., Crohn’s disease, ulcerative colitis, celiac disease); no use of antibiotics or probiotics in the preceding 3 months; no major dietary modifications in the past month; not pregnant or breastfeeding; not following any special diet; ability to provide stool samples and complete study questionnaires; and written informed consent.

Of the 20 healthy volunteers initially screened, 12 met all inclusion criteria and were enrolled.

**Figure 1:**
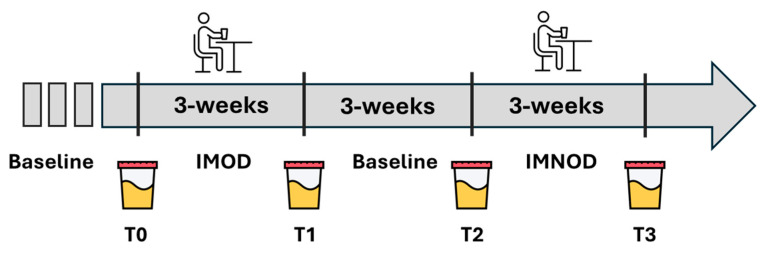
Experimental design. At the end of each 3 weeks, urine samples were collected. Abbreviations: IMNOD, Italian Mediterranean Non-Organic Diet; IMOD, Italian Mediterranean Organic Diet.

The same participants underwent both dietary intervention phases in a randomized sequence: one phase following the IMOD diet and the other the IMNOD diet. Each phase lasted 3 weeks and was separated by a 3-week washout period to minimize potential carryover effects (Figure 1).

Throughout the study, dietary adherence was assessed through regular dietary interviews. Biological samples were collected at baseline and at the end of each dietary phase to evaluate metabolic parameters. The crossover design allowed each participant to serve as their own control, thereby enhancing statistical power and reducing inter-individual variability.

### 2.2. Intervention Diets

The IMOD and IMNOD were designed according to the principles of the Italian Mediterranean Diet [18], ensuring comparable macronutrient composition across both conditions. The only difference between the two diets concerned the production method: participants in the IMOD phase consumed exclusively certified organic products, supplied free of charge by NaturaSì^®^, whereas those in the IMNOD phase consumed the same foods from conventional sources.

The two diets were matched for total energy intake and macronutrient and micronutrient composition. The total energy intake was personalized based on the subject’s metabolism.

Energy distribution was standardized as follows: 50–60% from carbohydrates, 15–20% from protein (approximately half of which was plant-based), and less than 30% from total fats, with saturated fats kept below 10% and cholesterol intake restricted to less than 300 mg/day. Dietary fiber intake was set at 30 g per day. Alcohol consumption was limited to a maximum of 100 mL of red wine per day. Weekly intake of animal-based products included four servings of fish, two servings of meat, and two servings of cheese. An animal-to-vegetable protein ratio of approximately 1:1 was maintained.

The baseline phase consisted of a 3-week period during which participants returned to their usual self-selected diet, free of nutritional counseling or imposed restrictions. This intermediate period also represented the pre-intervention condition at study entry. No specific recommendations were provided regarding portion sizes, food choices, or nutrient intake, and participants were instructed to maintain their habitual physical activity levels. This phase served both as a stabilization period between interventions and as the reference condition for assessing intervention-related metabolic changes.

### 2.3. Sample Preparation

Urine samples were initially thawed at room temperature, as they had been previously stored at −20 °C. After thawing, the homogeneity of each sample was assessed. In cases of inhomogeneity, the samples were vortexed to ensure proper mixing.

The pH of each sample was then measured using a calibrated pH meter equipped with a microelectrode. The pH values were recorded for all samples before further processing.

Subsequently, samples were centrifuged at 4000 rpm for 10 min at 4 °C. From each centrifuged sample, 540 μL of supernatant was transferred into a new Eppendorf tube. To this, 60 μL of a 1.5 M phosphate buffer (K_2_HPO_4_/NaH_2_PO_4_, pH 7.4), prepared in D_2_O and containing 80 mM sodium azide (to prevent bacterial growth) and 10 mM TSP (trimethylsilylpropanoic acid, used as a chemical shift reference), was added.

The resulting solution was vortexed to ensure homogeneity. A volume of 600 μL of the final mixture was transferred into 5 mm NMR tubes for spectral acquisition.

### 2.4. ^1^H-NMR Spectral Acquisition

All spectra were acquired using a Bruker Avance 700 MHz spectrometer (Billerica, MA, USA) employing the noesypr1d pulse sequence. The instrument was in a semi-automated acquisition mode (macro), enabling it to change and measure each sample automatically.

For each sample, the following acquisition parameters were used: temperature: 298 K; mixing time: 100 ms; spectral width: 12 ppm; acquisition time: 2 s; relaxation delay: 3 s; 128 scans; and 4 dummy scans. Each ^1^H-NMR spectrum required approximately 11 min to acquire.

### 2.5. Spectral Deconvolution

Post-acquisition spectra were processed using the Chenomx NMR Suite (Chenomx Inc., version 8.51, Edmonton, AB, Canada), specifically the Chenomx Processor module. Each Free Induction Decay (FID) file was imported, with the TSP concentration set at 1 mM and line broadening adjusted to 0.5 Hz. Manual phase and baseline corrections were performed for each spectrum. This software enables targeted profiling of ^1^H-NMR spectra by comparing experimental data with a curated library of reference spectra under the same acquisition conditions. Each urinary spectrum was analyzed by fitting known metabolite signatures to the observed peaks, ensuring accurate compound identification based on both chemical shift and multiplet pattern. A total of 42 urinary metabolites were confidently identified and quantified using this method.

### 2.6. Data Processing

#### 2.6.1. Delta Calculation

To determine the effect of the diet on the metabolic profile, percentage differences for each metabolite were calculated with respect to the baseline value, using the following formula:

$${∆}_{i,j}^{k}=\left(\frac{T_{i,j}^{2k-1}-T_{i,j}^{2k-2}}{T_{i,j}^{2k-2}}\right)\times 100$$

where, *k* = 1 denotes the percentage change between T1 and T0, and *k* = 2 between T3 and T2, for the *i*-th metabolite in the *j*-th sample.

#### 2.6.2. Data Scaling and Multivariate Analysis

For multivariate analysis, non-centered unit variation was applied to the delta matrices. Principal component analysis (PCA) was performed using SIMCA (UMetrics, Sartorius, version 18.0.1.507, Umea, Sweden).

## 3. Results

### 3.1. Subject Enrollment

In this longitudinal study, 12 healthy subjects were enrolled to follow different dietary regimens in order to investigate potential changes in their urinary metabolic profiles that might reflect the overall health status associated with a Mediterranean organic diet. Initially, urine samples were collected from all participants to establish a first baseline (T0). Subsequently, the subjects followed a strictly organic Italian Mediterranean diet (T1) for three weeks. After this period, a 3-week washout phase was implemented, during which participants resumed their habitual diet to restore their metabolic baseline (T2). Finally, the same individuals followed a second 3-week dietary intervention consisting of a non-organic Italian Mediterranean diet (T3) to evaluate metabolic alterations specifically associated with consuming organic versus non-organic products.

### 3.2. NMR Profiling Applied to Nutrition

Urinary samples collected at the different time points were analyzed by ^1^H-NMR spectroscopy to investigate potential changes in metabolite excretion associated with the dietary interventions. 42 metabolites were identified and quantified in most samples, with only 19% missing values. Metabolite quantification was performed through manual spectra deconvolution using a reference database of endogenous compounds (Table S1).

The identified metabolites cover a range of chemical and biochemical classes. Amino acids and their degradation products (e.g., valine, glycine, pyroglutamate, and 3-hydroxyisovalerate) indicate protein and nitrogen metabolism. Several organic acids reflect mitochondrial metabolic activity, such as citrate, succinate, fumarate, and acetate, key intermediates in the tricarboxylic acid (TCA) cycle. Sugars and polyols (e.g., glucose, arabinose, and erythritol) were also detected and may be linked to carbohydrate metabolism and dietary intake. Nitrogenous compounds reflect renal function and methylation processes, including creatinine, urea, dimethylamine, and trimethylamine N-oxide. Additionally, gut microbiota-derived metabolites (e.g., hippurate, 3-indoxylsulfate, phenylacetylglutamine, and 2-furoylglycine) were present, suggesting a microbial contribution to the urinary metabolome. Osmolytes such as betaine, carnitine, and taurine were also quantified, potentially associated with cellular stress response and osmoregulation.

### 3.3. Subsection Comparison to Physiological Reference Ranges Suggests Metabolic Stabilization Following Mediterranean Diet Intervention

**Figure 2:**
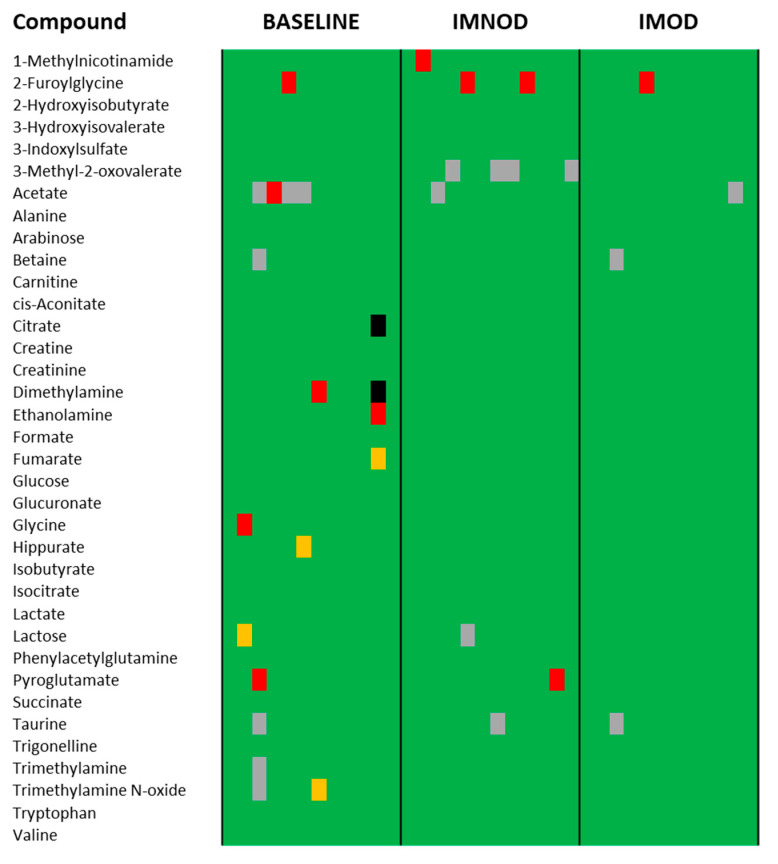
Heatmap showing the agreement between urinary metabolite concentrations (expressed in μM/mM of creatinine) and literature-based physiological reference ranges for each subject and each phase of the dietary protocol (baseline, IMNOD, and IMOD). Values in green lie within the normal range or exceed the limits by less than 5%. Values in yellow, red, and black indicate values higher than 1.35, 1.5, and 4 times the upper limit, respectively. Gray cells correspond to missing data. Abbreviations: IMNOD, Italian Mediterranean Non Organic Diet; IMOD, Italian Mediterranean Organic Diet.

As an initial assessment, the urinary concentrations of the 42 quantified metabolites were normalized to creatinine and compared against established physiological reference ranges (expressed in µM/mM of creatinine). Reference ranges were obtained from the Human Metabolome Database (HMDB) [25]. A heatmap was generated to visualize whether the observed values fell within or outside the normal range for each subject and time point (baseline, IMNOD, and IMOD) (Figure 2).

This analysis reveals that metabolite concentrations remained within the expected physiological ranges (green) in most cases. However, deviations were observed for several metabolites at baseline, including creatinine, dimethylamine, formate, glycine, hippurate, and pyroglutamate. These deviations were generally less frequent following both dietary interventions.

Some deviations persisted or emerged during the IMNOD phase, particularly for microbiota-associated metabolites such as 1-methylnicotinamide and 2-furoylglycine. Nonetheless, the overall proportion of out-of-range values was low, and the IMOD and IMNOD phases showed a relatively homogeneous distribution, suggesting that adherence to the dietary protocols may have contributed to stabilizing individual metabolic profiles.

### 3.4. Subsection Organic Foods Alter a Broader Range of Urinary Metabolites Compared to Non-Organic Foods

**Figure 3:**
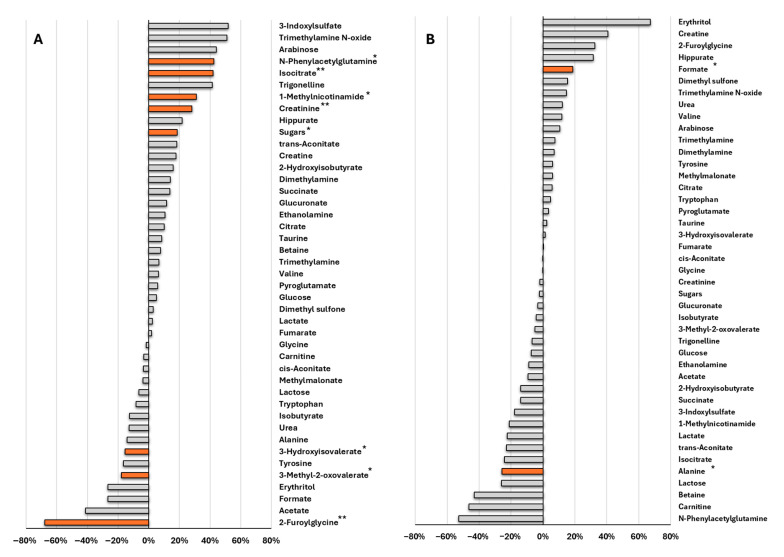
(**A**) Median percent change in metabolite levels after IMOD relative to baseline (T1 vs. T0), and (**B**) IMNOD relative to baseline (T3 vs. T2). Statistical significance was assessed using a paired *t*-test. Asterisks and orange bars indicate statistically significant changes (* *p* < 0.05; ** *p* < 0.01). Abbreviations: IMNOD, Italian Mediterranean Non Organic Diet; IMOD, Italian Mediterranean Organic Diet.

The analysis of median percent variation in urinary metabolites relative to baseline indicates that IMOD (Figure 3A) was associated with more statistically significant metabolic changes than IMNOD (Figure 3B).

A significant increase in isocitrate and trans-aconitate, both intermediates of the TCA cycle, was observed following the organic diet. In contrast, a marked decrease was found for formate and acetate, two low-molecular-weight carboxylic acids that are microbial fermentation products derived primarily from gut microbiota metabolism of dietary substrates. 2-Furoylglycine, a compound associated with microbial transformation of dietary aromatic components, was also significantly reduced. Additional microbiota-related metabolites, such as N-phenylacetylglutamine and 1-methylnicotinamide, showed significant increases, suggesting a diet-induced modulation of host-microbiota metabolic interactions. Within the amino acid and derivative class, a significant decrease in alanine, 3-methyl-2-oxovalerate, and tyrosine was detected, indicating changes in amino acid metabolism. A significant increase was also observed in trigonelline, a plant-derived metabolite commonly found in legumes and cereals.

In the IMNOD protocol, statistically significant changes were limited to formate and alanine (Figure 3B).

### 3.5. Comparative Effects of Organic vs. Non-Organic Diets on Metabolism

**Figure 4:**
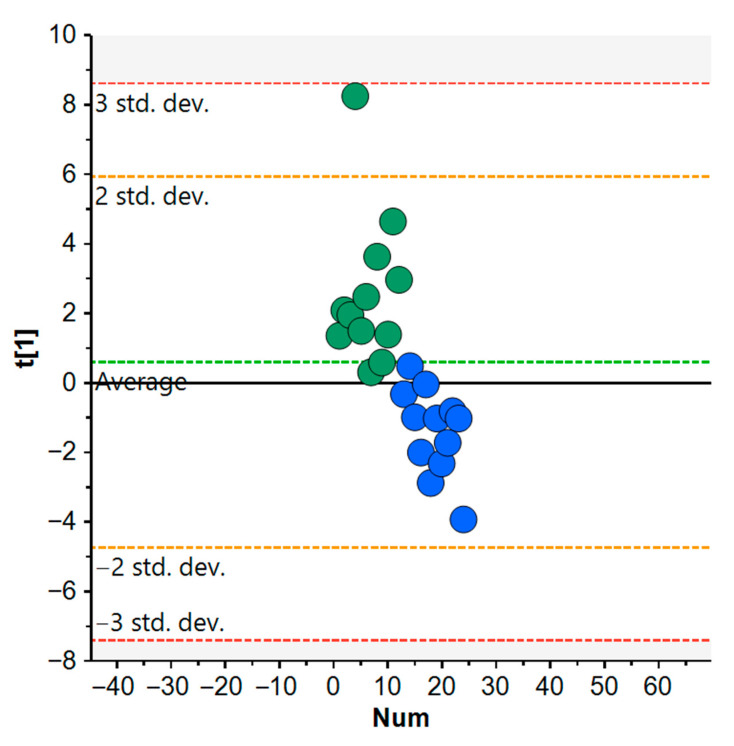
PCA score plot of urinary metabolites after dietary interventions. Each point represents an individual subject following either IMOD (green) or IMNOD (blue). The vertical axis (t[1]) indicates the first principal component, capturing the major source of variance across subjects. R^2^X = 0.17, Eigenvalue = 4.09, Q^2^ = −0.023. Abbreviations: IMNOD, Italian Mediterranean Non Organic Diet; IMOD, Italian Mediterranean Organic Diet.

PCA was employed to examine the overall variation in the urinary metabolome in response to IMOD and IMNOD (Figure 4).

The score plot revealed a clear separation between the two dietary groups along the first principal component, which accounted for 17% of the total variance. Post-intervention samples from the IMOD group (green) clustered toward positive t[1] values, whereas those from the IMNOD group (blue) clustered toward negative values. This distribution suggests that the two diets elicited distinct metabolic responses, with several urinary metabolites showing opposite excretion patterns depending on whether the diet included organic or non-organic food sources. The PCA results show a visual trend suggesting separation between the urinary metabolomic profiles of IMOD and IMNOD. However, the associated statistical parameters (R^2^X = 0.17; Q^2^ = −0.023) indicate that the predictive power is limited. Therefore, PCA in this context should primarily be viewed as an exploratory tool for identifying general patterns, rather than a method for making reliable classifications.

**Figure 5:**
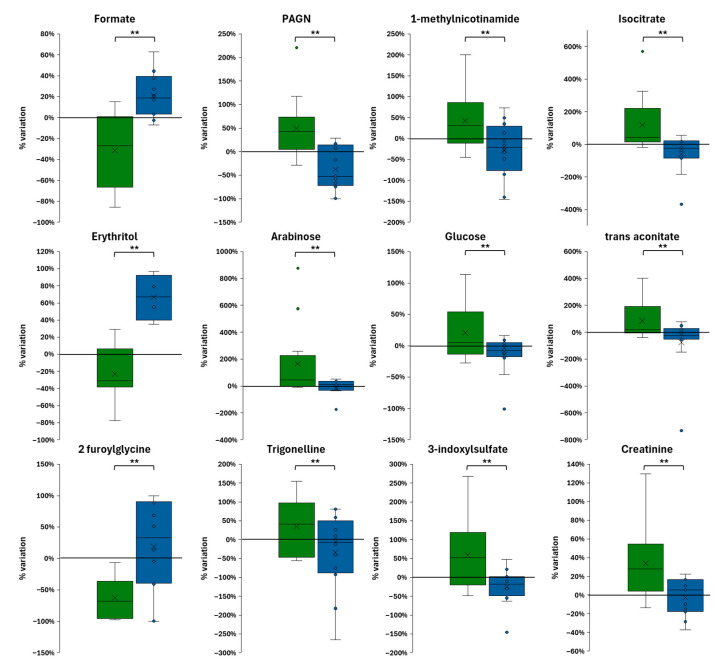
Box plots of percent changes in selected urinary metabolites after IMOD (green) and IMNOD (blue), relative to their respective baselines. Statistical significance was evaluated using a paired Student’s *t*-test (** *p* < 0.01). Abbreviations: IMNOD, Italian Mediterranean Non Organic Diet; IMOD, Italian Mediterranean Organic Diet.

These multivariate findings were consistent with the results of the univariate analysis of percent changes from baseline. Boxplots of selected metabolites (Figure 5) confirmed that isocitrate, trigonelline, arabinose, glucose, and trans-aconitate increased more markedly following the organic diet. In contrast, the same metabolites showed smaller or absent changes under the non-organic condition. 2-Furoylglycine showed a clear decrease after IMOD, with no comparable reduction in the IMNOD group. Creatinine increased in both diets but appeared slightly more elevated in the organic group. N-phenylacetylglutamine, 1-methylnicotinamide, 3-indoxylsulfate, carnitine, and succinate displayed modest or inconsistent variations and did not contribute significantly to the differentiation between the two dietary phases. The results support a specific metabolic response to the organic Mediterranean diet, characterized by enhanced excretion of carbohydrate- and plant-derived metabolites and a reduction in compounds linked to food processing or microbial proteolysis.

## 4. Discussion

In recent years, increasing attention has been given to the potential health implications of consuming organically produced foods, particularly within healthy dietary patterns such as the Mediterranean diet [26]. Organic farming systems restrict synthetic pesticides and fertilizers and have been associated with lower levels of environmental contaminants and higher concentrations of health-promoting compounds, including polyphenols [20]. Several observational studies have linked higher organic food consumption to favorable health outcomes, including lower incidence of metabolic syndrome, obesity, and certain cancers [27,28,29]. These effects have been attributed to reduced dietary exposure to synthetic agrochemicals and increased antioxidant and anti-inflammatory phytochemical intake. However, there remains a need for mechanistic studies that directly compare the metabolic impact of organic versus conventional diets under controlled conditions. In this context, metabolomics offers a powerful approach to assess systemic biochemical changes and to explore how food quality may influence metabolic health beyond macronutrient composition alone.

The present study investigated whether consuming organic foods as part of an IMOD induces different systemic metabolic responses compared to a non-organic version of the same diet (IMNOD). While the two diets were designed to be nutritionally equivalent regarding macronutrient composition and caloric content, they differed in food production methods, organic versus conventional agriculture. Using a targeted ^1^H-NMR metabolomics approach, urinary concentrations of 42 metabolites were measured in twelve healthy adult subjects before and after three weeks of each intervention, with a one-month washout in between. The longitudinal crossover design enabled the assessment of metabolic changes induced by IMOD and IMNOD within the same individuals, allowing for direct within-subject comparisons that control for inter-individual variability and isolate the specific metabolic impact of each dietary pattern.

Urinary metabolite concentrations were evaluated against established physiological reference ranges to assess whether either diet influenced the prevalence of out-of-range values. At baseline, a subset of metabolites, including formate, dimethylamine, hippurate, pyroglutamate, glycine, and creatinine, were found outside normal reference limits in several individuals. Following both IMOD and IMNOD interventions, these deviations were reduced. This suggests that adherence to a Mediterranean-style diet, regardless of whether it includes organic foods, stabilizes metabolic perturbations, bringing urinary profiles closer to physiological norms. This finding aligns with previous studies showing that the Mediterranean diet improves metabolic and inflammatory markers even in healthy individuals [30]. The stabilization effect observed here provides further evidence that dietary quality influences not only disease risk markers but also baseline metabolic homeostasis in healthy populations, thanks to an increased intake of fiber, polyphenols, and unsaturated fats [31,32].

A second layer of analysis focused on comparing metabolite levels following each dietary intervention and the individual baseline, offering a dynamic view of how each diet modulated metabolic profiles over time within the same subjects. Our results indicate that IMOD induced a broader and more statistically significant set of metabolic changes relative to baseline than IMNOD. The relatively limited metabolic impact observed following the IMNOD intervention may be attributed to participants already being accustomed to a Mediterranean dietary pattern, as the study was conducted in Italy [33].

Next, we analyzed the percentage variation of individual urinary metabolites relative to the baseline. We found that, when considering the overall profile, the direction of change was opposite between the IMOD and IMNOD interventions. This trend was clearly illustrated by the separation of the two dietary phases in the PCA, highlighting distinct global metabolic trajectories. This divergence may, at least in part, reflect specific characteristics of the study population. All participants belonged to a relatively homogeneous cohort with high educational and socioeconomic status, which is typically associated with greater awareness of healthy eating and a higher likelihood of organic food consumption, as supported by previous studies [28]. Since the IMNOD protocol completely excluded organic foods, while the IMOD was entirely organic, these interventions may have represented contrasting deviations from the participants’ usual dietary habits, which likely included a combination of conventional and organic Mediterranean foods, thus explaining the observed opposite metabolic responses.

For example, IMOD was associated with increased urinary excretion of isocitrate and trans-aconitate, both key intermediates of the TCA cycle. Beyond their classical role in energy metabolism, TCA cycle intermediates have been shown to modulate immune responses by supporting the metabolic demands of activated immune cells [34]. This suggests that the organic diet may contribute to a heightened immunometabolic state, potentially through increased bioavailability of micronutrients or phytochemicals that support immune cell function. At the same time, both formate and acetate, short-chain carboxylic acids commonly associated with bacterial fermentation, showed significant reductions in urinary levels following the IMOD intervention. This finding is particularly notable in light of previous work by Gupta et al. [35], who identified elevated urinary formate and acetate as metabolic signatures of bacterial overgrowth in urinary tract infections. While our study cohort did not present with infection, the observed reductions after the organic diet may reflect a decrease in bacterial fermentation activity, possibly due to modulations in gut microbiota composition or activity. Additional metabolites linked to gut microbial and host co-metabolism, such as N-phenylacetylglutamine and 1-methylnicotinamide [36], were significantly elevated after IMOD. These compounds reflect interactions between dietary inputs, microbial activity, and hepatic detoxification pathways.

The increased urinary levels of arabinose, glucose, and trigonelline following IMOD, metabolites often derived from plant-based foods, suggest that the organic diet may differ in bioactive compound composition or bioavailability, despite macronutrient similarities. These sugars are less concentrated in the urine of IMNOD subjects. Meanwhile, the reduction in 2-furoylglycine, a metabolite derived from food processing-related Maillard products, further supports the idea that organic food consumption may reduce dietary exposure to certain xenobiotics [37].

In summary, our findings suggest that the organic Mediterranean diet exerts a beneficial modulation of the MIIA, a functional interface between gut microbiota, host immune regulation, and inflammatory tone. This effect is reflected by enhanced host–microbiota metabolic crosstalk, lower urinary excretion of fermentation-derived and pro-inflammatory metabolites, and elevated levels of immune-supportive co-metabolites. These observations align with emerging evidence that host–microbiota communication plays a critical role in shaping systemic immune responses and metabolic resilience [38], particularly through pathways collectively known as the gut–immune–metabolism axis [39].

These findings provide novel mechanistic insight into how food quality in an Italian Mediterranean diet can influence systemic metabolism and immune-inflammatory balance. The distinct metabolic signatures observed after the organic Mediterranean diet highlight the potential of organically produced foods to modulate host–microbiota co-metabolism, reduce exposure to dietary xenobiotics, and support immunometabolic regulation.

Despite the novel insights provided by this study, several limitations should be acknowledged. First, the sample size was small, limiting the findings’ generalizability and the statistical power to detect subtle metabolic changes. Second, although participants followed the same dietary protocols during each intervention, their baseline dietary habits were probably homogeneous. Expanding a cohort of subjects with very different nutritional habits could improve the generalization of our results.

While the present study was conducted in a relatively homogeneous group of health-conscious individuals with higher socioeconomic status, who are more likely to consume already organic products, the health benefits observed should not be considered a privilege reserved for a select population. On the contrary, given the potential implications for long-term metabolic and immune health, these findings emphasize the importance of making organic, minimally processed foods more accessible across all social strata. Adopting such dietary patterns could yield substantial public health gains, particularly in preventing chronic inflammatory and metabolic conditions.

## 5. Conclusions

Understanding the impact of food production methods on human metabolism is critical to advancing dietary strategies for long-term health. While the Mediterranean Diet is well-established for its protective effects, the contribution of organic food quality within this dietary model has not been systematically explored through biochemical profiling.

In this study, we applied ^1^H-NMR urinary metabolomics in a randomized crossover design to evaluate the metabolic effects of two compositionally matched diets: the IMOD and IMNOD. Despite identical macronutrient content, IMOD induced a broader and more statistically significant shift in metabolic profiles, particularly involving host–microbiota co-metabolites, amino acids, and inflammatory mediators.

These findings led to the definition of the MIIA—Microbiota–Immune–Inflammation Axis—as a novel framework to describe the coordinated metabolic modulation observed under organic food intake conditions.

## Data Availability

This published article includes all of the data generated or analyzed during this study.

## Supplementary Materials

The following supporting information can be downloaded at: https://www.mdpi.com/article/10.3390/metabo15090571/s1, Table S1. Proton NMR chemical shifts (δ, ppm) corresponding to the 42 urinary metabolites analyzed in this study and used for their identification.

## Abbreviations

The following abbreviations are used in this manuscript:

3-week	3 weeks
FID	Free Induction Decay
IMNOD	Italian Mediterranean Non Organic Diet
IMOD	Italian Mediterranean Organic Diet
MIIA	Microbiota–Immune–Inflammation Axis
PCA	Principal Component Analysis
SCFAs	Short-Chain Fatty Acids
TCA	Tricarboxylic Acid
