# Supplementary material for: Urinary 1H-NMR Metabolomics Highlights MIIA (Microbiota–Immune–Inflammation Axis) Activation by Organic Mediterranean Diet
Source: Metabolites. 2025 Aug 26;15(9):571. doi: 10.3390/metabo15090571 (PMC12471874; doi:10.3390/metabo15090571)
Supplement: Supplementary file 1 [file metabolites-15-00571-s001.zip › metabolites-3808076-supplementary.pdf]

Table S1. Proton NMR chemical shifts (δ, ppm) corresponding to the 42 urinary metabolites analyzed in this study and used for their identification.

**Metabolite**	**δ [ppm]**
1-Methylnicotinamide	4.5, 8.2, 8.9, 9.0, 9.3
2-Furoylglycine	6.6
2-Hydroxyisobutyrate	1.3
3-Hydroxyisovalerate	1.3, 2.4
3-Indoxylsulfate	7.5, 7.7
3-Methyl-2-oxovalerate	0.9, 1.1
Acetate	1.9
Alanine	1.5
Arabinose	5.2
Betaine	3.3
Carnitine	3.2
cis-Aconitate	3.1, 5.8
Citrate	2.5, 2.7
Creatine	3.0, 3.9
Creatinine	3.0, 4.0
Dimethylamine	2.7
Dimethyl sulfone	3.1
Erythritol	3.6, 3.7, 3.8
Ethanolamine	3.1
Formate	8.5
Fumarate	6.5
Glucose	5.2
Glucuronate	5.2
Glycine	3.6
Hippurate	4.0, 7.5, 7.6, 7.8
Isobutyrate	1.1
Isocitrate	3
Lactate	1.2, 4.1
Lactose	5.2
Methylmalonate	1.2
Phenylacetylglutamine	7.3, 7.4
Pyroglutamate	2.4
Succinate	2.4
Taurine	3.3, 3.4
trans-Aconitate	6.6
Trigonelline	4.4, 8.1, 8.8, 9.1
Trimethylamine	2.9
Trimethylamine N-oxide	3.3
Tryptophan	7.7
Tyrosine	6.9, 7.2
Urea	5.8
Valine	1
